# 316. The Value of Monocyte-Lymphocyte Ratio, Neutrophil-Lymphocyte Ratio and Platelet-Lymphocyte Ratio in Predicting COVID-19 Mortality: A Single-Center Retrospective Study

**DOI:** 10.1093/ofid/ofac492.394

**Published:** 2022-12-15

**Authors:** Denise Marie J Nanas, Marjorie A Nolasco, Divina Cristy R Samin

**Affiliations:** Dr Paulino J Garcia Memorial Research and Medical Center, Cabanatuan, Nueva Ecija, Philippines; Dr Paulino J Garcia Memorial Research and Medical Center, Cabanatuan, Nueva Ecija, Philippines; Dr Paulino J Garcia Memorial Research and Medical Center, Cabanatuan, Nueva Ecija, Philippines

## Abstract

**Background:**

The quest for an easy yet effective and affordable tool in predicting mortality among COVID 19 patients is one of the major health concerns that should be addressed. In this study, we investigated the role of these hemogram derived ratios- Monocyte-Lymphocyte Ratio, Neutrophil-Lymphocyte Ratio and Platelet-LymphocyteRatio in predicting in-hospital COVID 19 Mortality. Objectives of this study aimed to determine the optimal cut-off value of each ratio and compare the values of each ratio in predicting mortality.

**Methods:**

A cross sectional retrospective study utilizing chart review of adult, COVID 19 confirmed patients admitted at Dr. Paulino J. Garcia Memorial Research and Medical Center from July 2020 to July 2021 were analyzed. Complete blood count taken within 24 hours of admission were recorded. The primary outcome was in-hospital mortality. The NLR, MLR and PLR values were computed and were stratified into two groups after determining the optimal cut-off from the receiver operating characteristic curve (ROC) curve.

**Results:**

A total of 226 adult Filipinos with confirmed COVID 19 infection were included. Patients who died had significantly higher mean age (p=0.004), lower mean hospital days (p=< 0.001) and significantly had a higher proportion of diabetes (p=< 0.027) as a co-morbidity. Significantly higher neutrophils (p=< 0.001), lower lymphocytes (p=< 0.001), higher NLR (p=< 0.001), higher MLR (p=0.002) and higher PLR (p=< 0.001) among non-survivors than survivors group. The optimal cutoff value of NLR to predict in-hospital mortality is ≥5.846 with 66.37% sensitivity and 60.18% specificity (AuROC curve of 0.695). The optimal cut off value of MLR is ≥0.4444 and PLR of ≥190.

**Figure:**
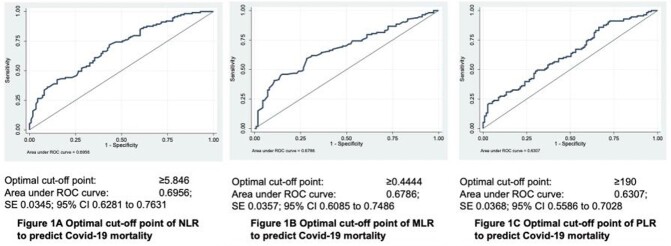

**Figure:**
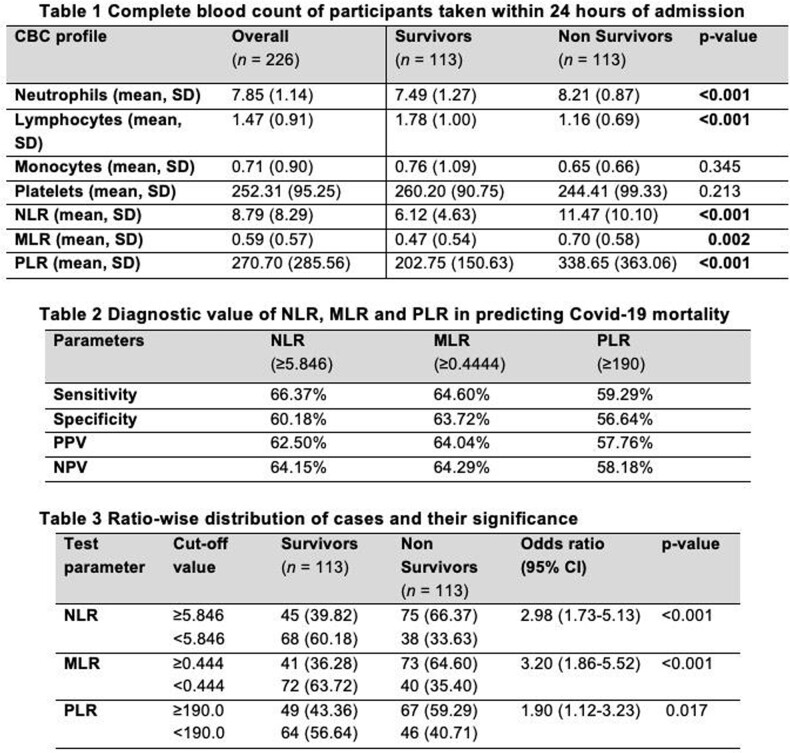

**Figure:**
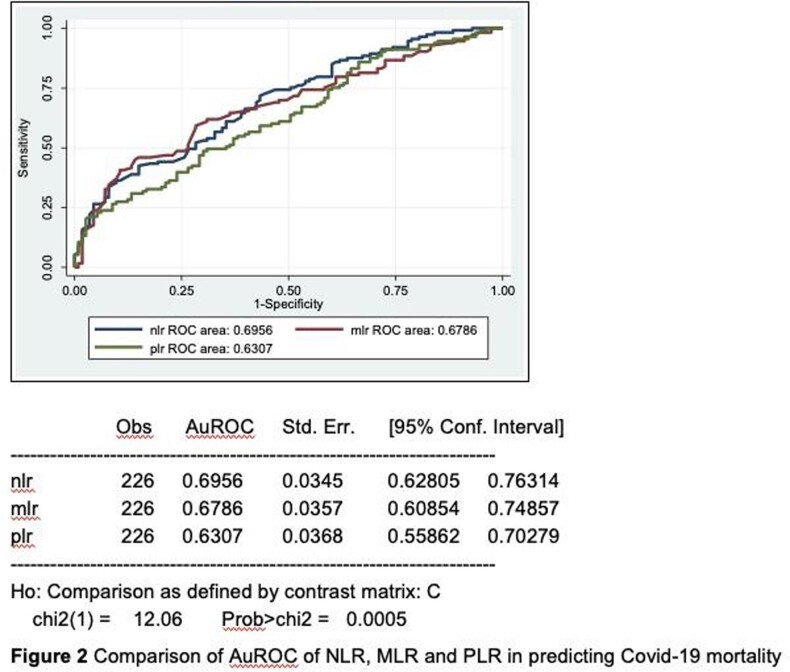

**Conclusion:**

NLR, MLR and PLR were noted to be consistently high among non-survivors of COVID 19 Infection. These are useful markers in predicting in-hospital mortality. NLR appears to be the best predictor of COVID-19 mortality of the three markers. However, its overall diagnostic accuracy is only considered acceptable at best.

**Disclosures:**

**All Authors**: No reported disclosures.

